# A new paradigm to induce mental stress: the Sing-a-Song Stress Test (SSST)

**DOI:** 10.3389/fnins.2014.00224

**Published:** 2014-07-29

**Authors:** Anne-Marie Brouwer, Maarten A. Hogervorst

**Affiliations:** TNO, Perceptual and Cognitive SystemsSoesterberg, Netherlands

**Keywords:** stress, arousal, paradigm, heart rate, skin conductance

## Abstract

We here introduce a new experimental paradigm to induce mental stress in a quick and easy way while adhering to ethical standards and controlling for potential confounds resulting from sensory input and body movements. In our Sing-a-Song Stress Test, participants are presented with neutral messages on a screen, interleaved with 1-min time intervals. The final message is that the participant should sing a song aloud after the interval has elapsed. Participants sit still during the whole procedure. We found that heart rate and skin conductance during the 1-min intervals following the sing-a-song stress message are substantially higher than during intervals following neutral messages. The order of magnitude of the rise is comparable to that achieved by the Trier Social Stress Test. Skin conductance increase correlates positively with experienced stress level as reported by participants. We also simulated stress detection in real time. When using both skin conductance and heart rate, stress is detected for 18 out of 20 participants, approximately 10 s after onset of the sing-a-song message. In conclusion, the Sing-a-Song Stress Test provides a quick, easy, controlled and potent way to induce mental stress and could be helpful in studies ranging from examining physiological effects of mental stress to evaluating interventions to reduce stress.

## Introduction

When studying physiological effects of sudden arousing and negative emotional events, it is desirable to induce a considerable amount of emotional stress in an easy, controlled and efficient manner, while respecting ethical standards with regard to experimental participants. A range of protocols in this direction has been described in the literature. Kreibig (2010) lists experimental paradigms of 134 studies on effects of emotion on autonomic nervous system activity. With respect to the negative emotions fear and anxiety, paradigms that have been used include watching film clips (Eisenberg et al., 1988), viewing pictures from the standardized International Affective Picture System (IAPS) by Lang et al. (2005) (Ritz et al., 2005), listening to sounds from the standardized International Affective Digitized Sound system (IADS) by Bradley and Lang (2007) (Brouwer et al., 2013), listening to music (Krumhansl, 1997), expecting an electrical shock (Bloom and Trautt, 1977), mental imagery (Van Diest et al., 2006), negative events in a gaming context (Brouwer et al., 2011), recall of personal emotional events and directed facial expression (Ekman et al., 1983). The paradigm that has become the worldwide standard for inducing psychological stress is the Trier Social Stress Test (TSST—Kirschbaum et al., 1993). In this paradigm, participants are asked to take over the role of a job applicant. They are informed that they will be giving a speech to a “selection committee” consisting of three people, and that their speech performance will be video and audio recorded for analysis. At the start of the procedure, participants get to see the business-like equipped test room with the committee, wearing white coats. During a stress anticipation interval of 10 min, participants prepare a speech that should convince the selection committee that they are the perfect candidate for a job. Participants are provided with paper and pencil to help them prepare. Following the stress anticipation interval, they deliver their speech standing in front of the selection committee. The members of the committee respond in a standardized, non-emphatic way. After 5 min of free speech, the participant is instructed to perform a serial subtraction task aloud, having to start over again on each mistake. In a later version, the paradigm has been adapted slightly to involve two to three people in the selection committee and only 3 min of preparation time (Kudielka et al., 2007).

The TSST is considered the best available paradigm to reliably elicit strong neuroendocrine stress responses (Dickerson and Kemeny, 2004). Responses of the sympathetic nervous system are also strong in comparison to other paradigms. Heart rate increases in the order of 17 bpm (Kirschbaum et al., 1993; 20 bpm in a TSST for groups: von Dawans et al., 2011) when comparing a baseline measurement to a measurement taken at the end of the 10-min preparation interval. In a public speaking task that is comparable to the TSST but that made use of a virtual rather than a real audience (Westenberg et al., 2009), heart rate increased with about 10 bpm and skin conductance increased with approximately 2 μS. In their TSST review paper, Kudielka et al. (2007) mention a heart rate increase of 15–25 to the psychosocial stressor. It is unclear though which heart rate values are compared to arrive at this estimate. It probably involves heart rate during performance of the task, which is arguably the time that the most stress is experienced but which is also confounded by standing and talking such that part of the increase in heart rate is likely due to physical activity. As a rough comparison to physiological effects found using the TSST, viewing arousing pictures with negative valence usually elicits short rises in skin conductance of at most a few tenths of μS (Codispoti et al., 2001; Codispoti and De Cesarei, 2007) while heart rate decelerates for about one or at most a few bpm (Codispoti and De Cesarei, 2007; Brouwer et al., 2013).

The stress elicited by the TSST is multi-facetted. Participants perform cognitive tasks while preparing and delivering a speech, they are standing upright and talking, but the element that is considered to be mainly responsible for at least the strong neuroendocrine stress responses is social-evaluative threat (Dickerson and Kemeny, 2004). Social-evaluative threat occurs when an important aspect of the self-identity is, or could be negatively judged by others.

While the TSST elicits reliable stress responses and is a well-studied paradigm, it is a relatively complicated procedure geared to examining neuroendocrine responses. We present an easy, short and effective stress-inducing paradigm that is geared toward examining stress responses controlled by the sympathetic nervous system such as skin conductance and heart rate. These stress responses typically occur faster than neuroendocrine responses. However, they can also be strongly affected by posture or body movements such as speech, so it is important to keep these constant across stress levels. In our paradigm, participants are presented with neutral messages on a screen, interleaved with 1-min time intervals. The final message is that the participant should sing a song aloud after the interval has elapsed. Participants sit still during the whole procedure. Skin conductance and heart rate are compared between time intervals that follow neutral messages and the one that follows presentation of sing-a-song “stress” message. In contrast to the TSST and a number of other stress inducing paradigms, both body movements and sensory input are kept constant while only mental stress is varied. This ensures that effects of the stress message on skin conductance and heart rate can only be attributed to psychological processes, and are not caused or partly caused by an increase in visual input or by the fact that the individual starts moving or talking.

In accordance to the stress inducing potency of social-evaluative threat, earlier studies indicate that anticipating and watching oneself singing with an audience, does indeed elicit emotional stress. In a study by Harris (2001) participants sang “Star Spangled Banner” while being recorded on video. After singing, there was a 10-min break followed by a 6-min relaxation period during which physiological baseline measures were taken. Then, the participant watched the video tape together with the experimenter and two confederates. No instructions were given to the participants on how to behave during this viewing interval. During the first minute of the viewing interval, heart rate was 4.5 (first study) or 3 (second study) bpm higher than during the baseline measurement. Changes in heart rate were significantly correlated with subjective ratings of feeling calm (*r* = −0.43) and feeling embarrassed (*r* = 0.38). Hofmann et al. (2006) investigated autonomic correlates of social anxiety and embarrassment in shy and non-shy individuals. They informed their participants upon arrival in the lab that they would be asked to give a speech and to sing in front of a video camera. Their results indicated that anticipating singing results in a stronger increase of heart rate (compared to an eyes-closed baseline) than either anticipating giving a speech or watching videos of the performance together with confederates. Average heart rate increase was 11 bpm, and skin conductance increased with approximately 4 μS.

The main aim of the present study was to examine the potency of our Sing-a-Song Stress Test to elicit stress-related increases in heart rate and skin conductance. On the one hand, we expected the test to be effective given relatively strong effects of previous studies on social evaluative threat and singing, on the other hand, effect sizes may have been expected to be small since we excluded effects of confounds that possibly contributed to effects in other studies. We checked whether eventual increases could be detected by a change detection algorithm on the level of an individual participant, simulating an online stress detection algorithm in a situation that a participant is sitting still. Ultimately, we are interested in online and real-life detection of sudden stress based on physiological signals—if possible without attaching sensors to the body. Wieringa et al. (2005) showed that it is possible to recover heart rate from no-contact recorded images. Poh et al. (2010) described a robust algorithm to determine heart rate from varying color of a face as recorded by a camera. A similar algorithm has been exploited in Vital Signs Camera technology by Philips. To compare the performance of this technology to heart rate as measured using traditional electrocardiography (ECG), we measured heart rate using ECG and a camera simultaneously. A previous experiment indicated that heart rate based on camera images is very similar to heart rate extracted from ECG when averaging across 2 min intervals (Hogervorst et al., 2013).

## Materials and methods

### Participants

Twenty-five participants (15 female, 10 male) took part in the experiment. They were between 19 and 50 years old with a mean age of 30 and a standard deviation of 11 years. None of them suffered from a heart disease or took drugs that affect heart rate, as verified through a questionnaire. Participants received a monetary reward to make up for their travel and time. The study is in accordance with the Declaration of Helsinki and has been approved of by the local ethics committee. All participants signed an informed consent form prior to taking part in the experiment.

### Materials

#### Stimuli

We selected nine phrases that were approximately of the same length and structure as the 10th sentence which was the following (translated into English): “Task: start singing a song aloud when the counter reaches zero. Keep sitting still until that moment.” Since we did not want the other nine phrases to elicit stress, we picked neutral phrases from the Dutch Wikipedia site about vacuum cleaners. A translated example is “Phrase: the first vacuum cleaner was constructed by Sweep Company. This was in 1907 and the device was called hoover.”

#### ECG and skin conductance

To record ECG, we used self-adhesive Kendall Neonatal ECG electrodes (TYCO healthcare, Neustadt, Germany). The ECG channel electrode was placed at the sixth left intercostal space (midclavicular line); the reference electrode was placed at the first right intercostal space (midclavicular line); the ECG ground electrode was placed at the sixth right intercostal space (midclavicular line). Recording frequency was 256 Hz. To record skin conductance, we used the g.GSRsensor2 from G.tec Medical engineering (G.tec GmbH, Schiedlberg, Austria). Electrodes were applied to the fingertips of the index finger and the middle finger. Physiological data was processed by a G.tec USB Biosignal amplifier.

#### Vital signs camera

Heart rate was also determined using a camera and Philips Vital Signs camera software. The camera was an IDS UI-2220SE-C-HQ (uEye SE) 12-bit color camera, recording raw RGB-video, 8-bit per channel at 20 Hz and 768 × 576 pixels. The Vital Signs camera software requires the user to initially indicate a skin location by drawing a rectangle around a suitable face region as displayed on a monitor. This region is then automatically tracked. The Vital Signs camera software derives a measure of heart rate from subtle variations in skin color. Each measure is based on a window of 25.6 s and the resulting heart frequency is reported with a resolution of 0.039 Hz or 2.34 bmp at an output frequency of 20 Hz.

### Procedure

Participants were not given any instructions prior to the study e.g., with respect to eating and drinking or physical activity prior to coming into the lab. The participant, as well as two confederate “participants” (one male and one female), were picked up by the experimental leader from the waiting area. In the experimental room, the experimental leader explained that they would be sitting in turn behind the monitor while heart rate and skin conductance are monitored and they are filmed by a camera. The task description was to sit as still as possible and silently read the messages that appear on the monitor, interchanged by a counter counting down from 60 to 0 s. They were told that one of the messages could entail a task that they then needed to carry out after the subsequent counter reached 0. Participants were not told that the experiment was about stress or involved singing. Subsequently, the participant and the confederates filled out the informed consent form as well as a questionnaire with general questions about age, use of cigarettes, coffee and alcohol, physical activity and cardiovascular health. The experimental leader then appointed the real participant as the one to start, seated him or her behind the monitor and then attached the physiological sensors. The monitor was in between the participant and the confederates, who were sitting on chairs facing a wall such that the angle between their gaze and that of the participant was 90 degrees. After the experiment, participants were asked whether they had believed that the confederates were experimental participants. They were also asked to indicate the stress they experienced during the minute before singing on a scale of 1 (not stressed at all) to 10 (extremely stressed). The experimental room did not have windows to the outside. Lighting was constant office lighting.

### Analysis

#### ECG and skin conductance

The ECG signal was filtered by the g-tec amplifier with a 0.5–100 Hz band pass filter, and afterwards by a 2.5 Hz high-pass 2-sided Butterworth filter. R wave to R wave interval (RRI, the interval between successive heart beats) was extracted from this signal. Outliers resulting from the RRI derivation were determined by calculating differences from a moving median as calculated over 30 samples. Values outside the 5–95% quantile range were removed. For each participant, we then determined the mean RRI of each of the 1-min count-down intervals (blocks), and mean RRI was converted to HR (bpm). Also, mean skin conductance level was calculated over the 1-min blocks.

#### Statistical analysis

In order to test whether heart rate and skin conductance show a sudden increase after presentation of the sing-a-song sentence, we performed paired *t*-tests on values of heart rate and skin conductance averaged over the 1-min blocks after offset of the 9th (vacuum cleaner) message and the 10th (sing-a-song) message, for each participant. Correlations are computed between subjective stress ratings and differences in heart rate between the 9th and the 10th block as well as between subjective stress ratings and differences in skin conductance between the 9th and the 10th block. Positive values indicate higher values in the 10th than in the 9th block.

#### Stress detector

By designing a set of stress detectors based on detecting changes in state, we wanted to get an impression to what extent the onset of the sing-a-song message could be detected for a single individual and in real time. Note that it was not our goal to design the most optimal detector. Performance of these detectors can thus be considered to be the lower bound to performance under the circumstances tested.

#### Basic change detection algorithm

The “normal” state of the signal was described by calculating the average and standard deviation of the signal during blocks 1–9 of the experiment, for each of the participants separately. Next, a normalized signal, akin to the *z*-score, was computed by subtracting the mean signal and dividing this difference by the standard deviation. A threshold was set above which a signal was considered to be deviating from normal, therewith signaling a change of state. Here, a change was assumed to correspond to the onset of stress. For each of the types of signals (as used in different detector types as described below) a suitable threshold was chosen, such that a change in physiological state was most often detected in block 10 and least often in other blocks (false alarms).

#### Detector types

Detectors were designed that made use of the various types of information available. Two detectors were based on heart rate only: one was based on heart rate derived from the ECG signal and one on heart rate derived from the camera data. The stress detection threshold for these detectors was set to a value of 4.

For skin conductance we used two different types of detectors. One detector used the raw signal with a threshold value of 6. Another detector used a filtered version of skin conductance. Filtering was done by subtracting the mean over the interval starting at 33 s and ending 3 s before the current moment from the mean over the last 3 s. This filter acts as an edge detector and eliminates slow variations in the skin conductance. The filtered signal was used as signal input to the basic change detection algorithm. We used a threshold value of 6 for this skin conductance edge detector (i.e., the same as for the skin conductance detector based on the raw signal). The skin conductance edge detector turned out to perform better than the detector based on the raw signal due to the existence of slow variations in the skin conductance level.

Finally, a detector was constructed in which the skin conductance edge output and ECG heart rate were combined. Both signals were converted to a normalized *z*-score before they were combined by calculating the square root over the sum of squares and dividing by the square root of 2: *z* = sqrt(*x*^2^ + *y*^2^)/sqrt(2) (where *x* is the *z*-score of the skin conductance signal, *y* is the *z*-score of heart rate, and *z* is the combined *z*-score). In correspondence with the individual thresholds (6 and 4) the threshold for the combined signal was set to 5.

## Results

### General

All 25 participants started singing. Three participants accidentally started to sing too early, that is, before the counter had reached zero. After the experiment had ended, four participants stated that they had doubted that the confederates were real participants. None of them was a participant who started singing early. The data of the three early singing participants are excluded from further analyses to prevent confounding the mental stress interval with body movements.

### Heart rate response

**Figure 1:**
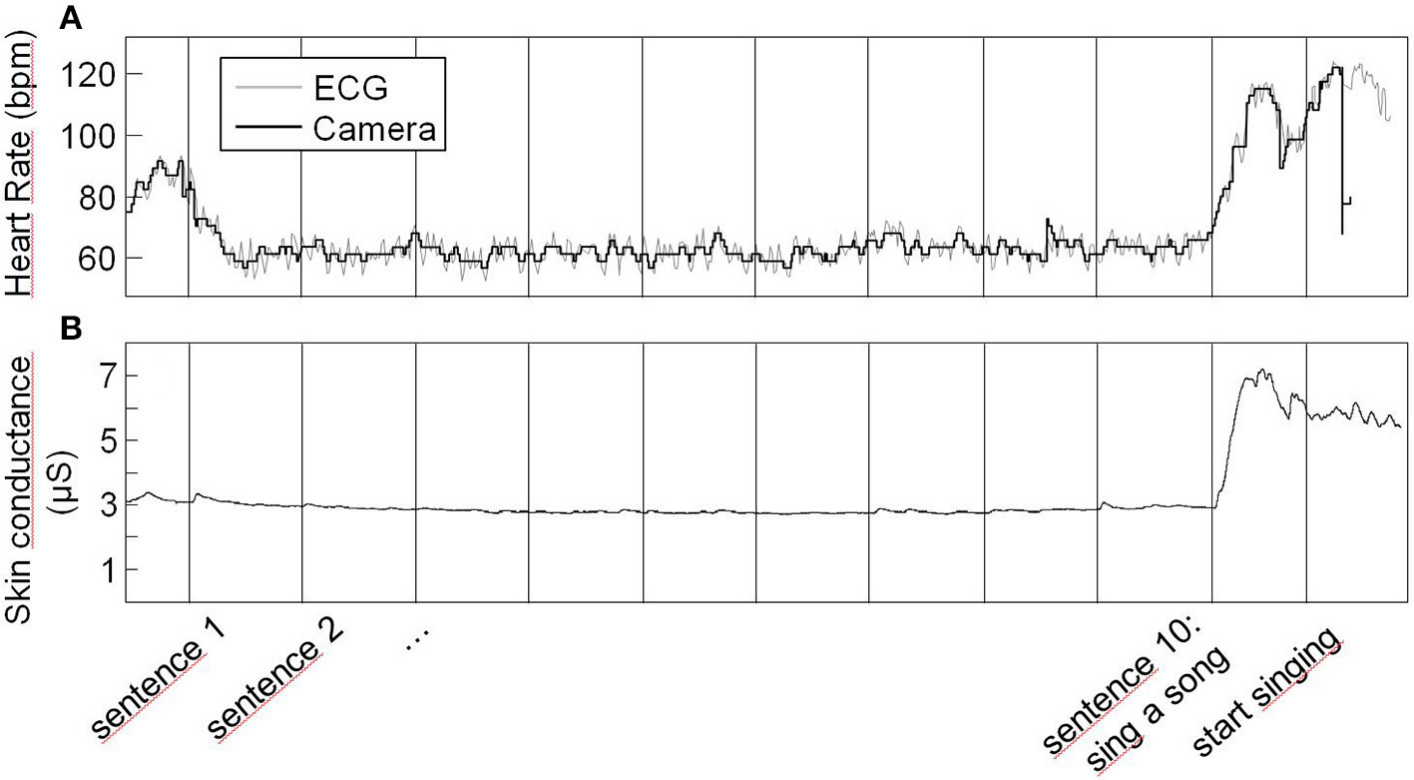
**Heart rate (A) and skin conductance (B) of an example participant across the whole experiment**. Vertical lines indicate the sentence onsets except for the very last one that corresponds to singing onset. Heart rate depicted by the gray line is heart rate derived from ECG; heart rate depicted by the black line is heart rate derived from the camera.

**Figure 2:**
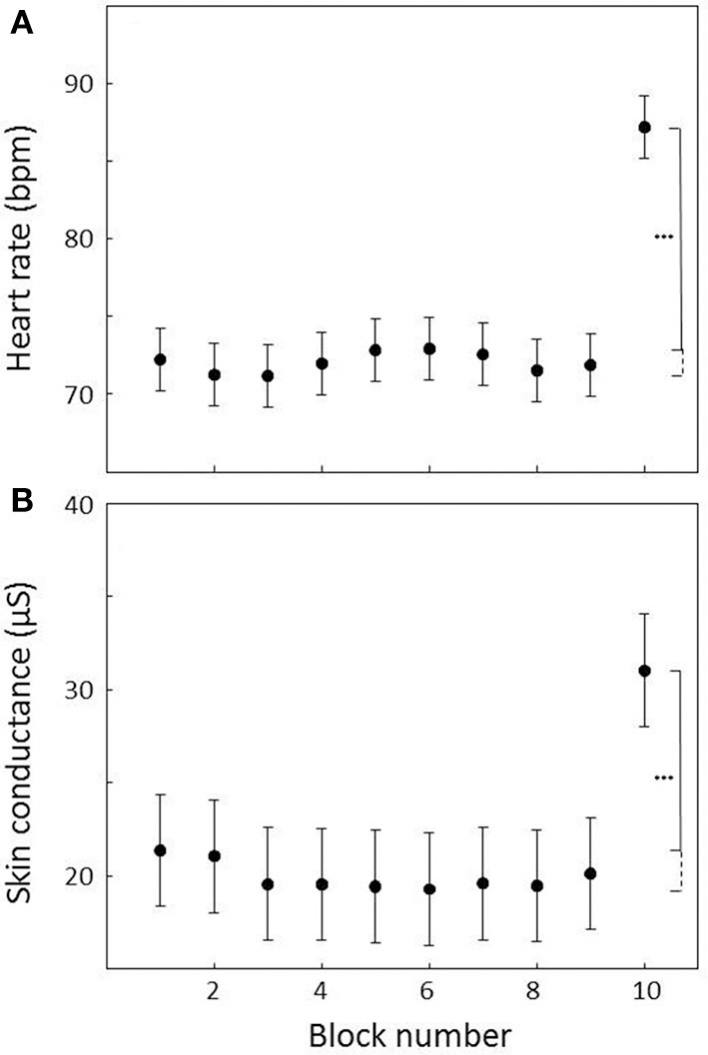
**Heart rate (A) and skin conductance (B) averaged across 1-min count-down blocks across all subjects**. Block 10 is the count-down block following the sing-a-song sentence. Error bars indicate standard errors of the mean. Stars indicate that heart rate and skin conductance are significantly higher in block 10 compared to all other blocks at an alpha level of 0.001.

Heart rate responses to the presentation of the sing-a-song sentence were generally very strong as indicated by the data of an example participant in Figure 1A. For every participant, heart rate was on average higher in the 10th than in the 9th block. Figure 2A indicates the heart rate for each of the 10 blocks averaged across participants. A paired *t*-test indicates a significant difference between the 9th and the 10th block (*t*_21_ = 6.21, *p* < 0.001, mean difference: 15.3 bpm, Cohen's d: 1.63, paired *t*-tests comparing the 10th block to all other blocks resulted in *p*-values < 0.001 as well).

### Skin conductance response

Like heart rate responses, skin conductance responses to the presentation of the sing-a-song sentence were generally very strong. Data of an example participant is presented in Figure 1B. For two of the participants, skin conductance recording failed. For all of the 20 remaining participants, skin conductance was on average higher in the 10th than in the 9th block. Figure 2B indicates the average skin conductance in the different blocks. A paired *t*-test indicates a significant difference between the 9th and the 10th block [*t*_(19)_ = 5.37, *p* < 0.001, mean difference: 10.9 μS, Cohen's d: 0.81, paired *t*-tests comparing the 10th block to all other blocks resulted in *p*-values < 0.001 as well].

### Subjective ratings

**Figure 3:**
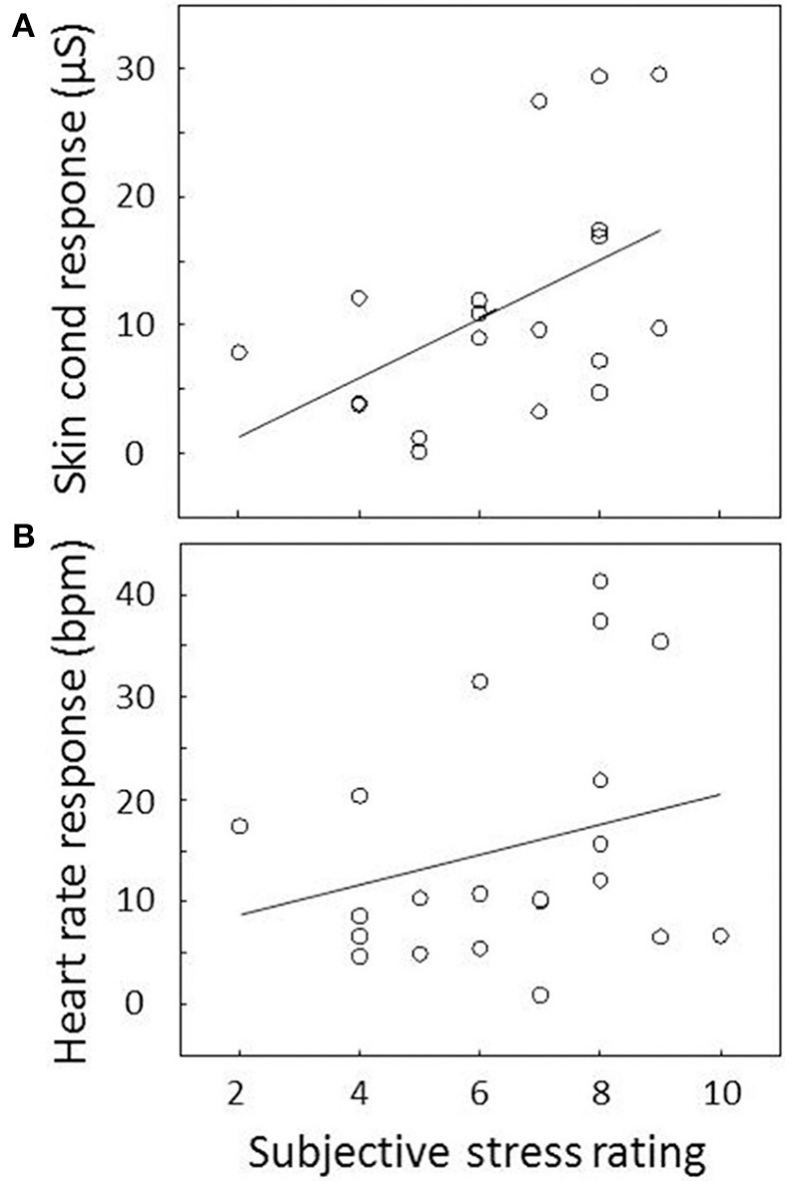
**Association between subjective stress and (A) skin conductance and (B) heart rate response**. Responses are determined by the difference in average skin conductance level or heart rate between the 9th and the 10th block and plotted against subjective stress rating for each participant. The correlation between subjective stress and skin conductance response was significant; the correlation between subjective stress and heart rate response was not. The plotted lines are linear regression lines.

The subjective rating of experienced stress was missing for one out of 22 participants. Ratings vary from 2 to 10 with a mean of 6.4 and a standard deviation of 2.1. As indicated in Figure 3A, subjective ratings correlated with skin conductance response (i.e., the difference in mean skin conductance level between the 9th and the 10th block: *r* = 0.50, *p* = 0.03). We did not find a significant correlation between subjective ratings and heart rate response (Figure 3B; *r* = 0.26, *p* = 0.26).

### Vital signs camera system

**Figure 4:**
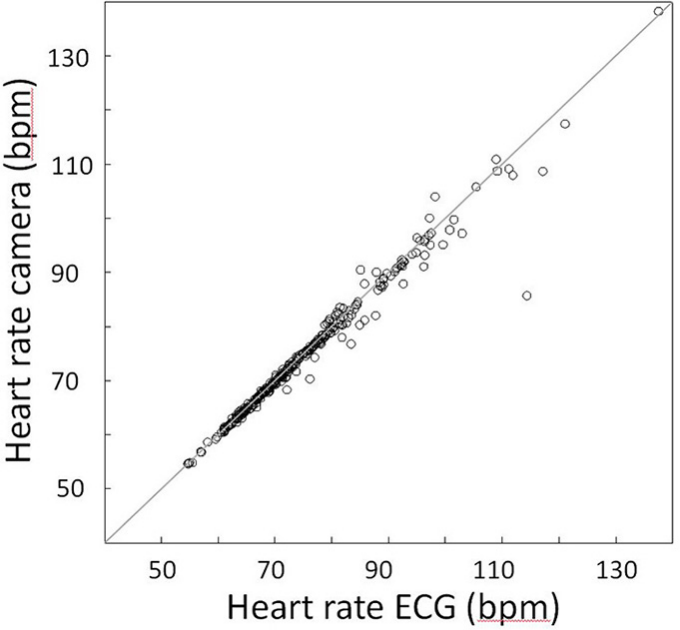
**Association between heart rate as determined by the camera and heart rate as determined by ECG**. The diagonal line is the unity line. Each symbol represents the average heart rate as derived from ECG and the camera for one 1-min block for one participant.

There is a strong correspondence between heart rate as determined by the Vital Signs camera system and ECG. The example data in Figure 1A show that while the high frequency heart rate variability cannot be closely followed due to limited resolution, the heart rate as given by the camera system nicely follows the ECG data. The scatter plot presented in Figure 4 represents average heart rate values for each of the 25 participants and each 1-min block as based on the camera and ECG. On average, ECG indicates a heart rate that is 0.55 bpm faster (*SD* = 2.22) than the Vital Signs Camera system. The median indicates a 0.25 bpm faster heart rate. The mean absolute difference is 0.92 bpm (*SD* = 2.09). The median absolute difference is 0.38 bpm. 77% of the heart rate values as measured by the camera is within 1 bpm difference from ECG; 90% is within 2 bpm difference.

### Stress detector

Table 1 shows the results of the various stress detectors for 20 participants since data of the three participants that started to sing early and the data of the two participants with failed skin conductance recording were excluded. Shown are the number of participants for which a state change was detected in block 10 (“Detections”) and the total number of detected state changes during blocks 1–9 (false alarms: “FAs”) over all participants. The false alarms often occurred at the start of the experiment and seemed at least partly be due to signal recording artifacts. When false alarms occurring during the first 20 s of the experiment were not counted, this resulted in a 60% reduction of false alarms as indicated in the table. The last two columns show the mean and median detection times (DTs), indicating the time between the start of block 10 and detection. For determining detection times, we only used blocks in which a state change was detected. These detection times are quite short, especially considering that the participant and his/her physiological system need some time to react to the stimulus.

**Table 1:** **Threshold settings and results for the different stress detectors**.

**Detector**	**Threshold**	**Detections**	**FAs**	**FAs after 20 s**	**Mean *DT***	**Median *DT***
Skin conductance raw	6	15 (75%)	0	0	15.3	6.8
Skin conductance edge	6	17 (85%)	7	2	7.7	6.7
Heart rate camera	4	16 (80%)	13	7	23.3	26.0
Heart rate ECG	4	17 (85%)	3	1	24.3	22.1
Heart rate ECG + skin conductance edge	5	18 (90%)	2	0	11.1	8.1

*“Detections” are the number (and %) of participants out of 20 for which stress was detected in the 10th block. “FAs” are the total number of false alarms (“stress detections” in block 1–9). “FAs after 20 s” represents the same but after excluding the first 20 s of block 1. “Mean DT” and “Median DT” (detection time in s) indicate when stress was detected relative to the onset of the sing-a-song sentence*.

Of the single sensor detectors the skin conductance edge detector performs best, detecting a change in the 10th block for 85% of the participants. The detector that uses the raw skin conductance signal shows the lowest detection performance (75% of the participants) indicating that preprocessing is required to compensate for the slow variations in the raw signal.

The detector that uses heart rate as derived from ECG seems to perform better than the one based on the camera in terms of the number of detections and the number of false alarms. Whereas both signals are largely the same, the resolution of the camera signal is limited resulting in more noise.

The detector that uses a combination of heart rate (from ECG) and filtered skin conductance performs best, with a detection rate of 90% (18 out of 20 subjects) and only two false alarms that were both found during the first 20 s of the experiment. The missed detections by the 5 different detectors originate from 8 participants, labeled “A” through “H” in Table 2. This table shows the overlap of participants with missed detections across the different detectors. For one of the participants (“A”), none of the detectors detected a state change in block 10. It is possible that this participant simply did not experience stress. Participants B and C did not show a detectable skin conductance response to the stress message while heart rate did show a response. The converse is true for participants D and E. More noisy variants of skin conductance (i.e., the raw data version) and heart rate (i.e., the camera version) resulted in below detection effects for three participants for one or the other signal (participant F, G, and H).

**Table 2:** **Participants without detected stress response for at least one detector**.

**Detector**	**Participant**							
	**A**	**B**	**C**	**D**	**E**	**F**	**G**	**H**
Skin conductance raw	×	×	×			×	×	
Skin conductance edge	×	×	×					
Heart rate camera	×			×	×			×
Heart rate ECG	×			×	×			
Heart rate ECG + skin conductance edge	×		×					

The missed detections per detector equal the number of detections as presented in Table 1 subtracted from 20 (the number of included participants).

## Discussion

### The sing-a-song stress test

The Sing-a-Song Stress Test proved to be an effective way to induce stress: rises in heart rate and skin conductance across 1 min, or even a fraction thereof, were in the same order as those elicited by the TSST viewed over 10 min. Rises in heart rate and skin conductance responses were an order of magnitude larger than those generally reported to be elicited by perceptual stimuli. Most subjective stress ratings reflected moderate to strong experienced stress, and individual subjective ratings correlated with rises in skin conductance. The same trend was observed for heart rate. As opposed to the TSST, our paradigm features a sudden stressor with known onset which makes it suitable to investigate physiological responses in short time windows. This paradigm also offers a clear within-subject baseline that controls for sensory input and body movement, and that is close in time to the stress interval (i.e., the 9th “neutral” count-down block as compared to the 10th “stress” count-down block). Since the paradigm only involves a passive audience of two confederates who are, during the experiment, not directly in the line of sight of the participant, possible effects of confederates behaving differently toward different participants are minimized. The limited role of the confederates would also potentially enable the use of different confederates within one project. The Sing-a-Song Stress procedure is short, with a complete experiment lasting about 11 min. The only equipment that is needed is a computer and a screen presenting messages at fixed time intervals. The limited number and role of the confederates, the short procedure and the fact that no special equipment is needed make the paradigm low-cost. Since participants do not move and are in front of a monitor rather than in front of a real-life audience, the test could in principle be applied in an MRI scanner (as also addressed in Quaedflieg et al., 2013). Stress induction paradigms, such as the Sing-a-Song Stress Test, can be useful in a range of stress-related studies (Kudielka et al., 2007). Examples include validating stress response sensitivity of physiological sensors or signal processing algorithms, evaluating interventions to reduce stress or examining the association between personality characteristics and stress responses.

### Limitations of the paradigm, possible improvements and future experiments

The instruction of singing after the 10th count-down interval was not followed by three out of 25 participants. They started singing earlier than that and we had to exclude these data to prevent confounding the mental stress interval with body movements. If we had included data of these participants, mean heart rate increase would still have been 15.3 bpm (as is the average hart rate increase without these participants), while the skin conductance increase would on average have been larger (12.6 μS rather than 10.9 μS). This stronger response would both be consistent with an effect of movement or a different kind of breathing, and of early singers being more stressed than the other participants which would arguably be consistent with the fact that they were not able to follow the instruction.

The Sing-a-Song Stress Test induces stress by asking participants to sing a song after a designated interval is over. While physiological effects can only be due to mental processes, we do not know exactly which mental processes contribute to the observed effects, similar to the TSST. Both the TSST and the Sing-a-Song Stress Test involve mental workload besides social anxiety and (anticipated) embarrassment. The cognitive task of our participants was arguably easier than preparing a speech, but they did need to choose a song to sing. Increasing workload (therewith probably also increasing stress) has been found to be associated with increasing heart rate and skin conductance. In an experiment that varied workload while keeping perceptual input and movement constant, heart rate was about 4 bpm higher and skin conductance level about 1.5 μS higher in a quite difficult high workload condition compared to a very easy, low workload condition (Brouwer et al., 2014). While it may be impossible to completely disentangle mental processes such as workload and more emotion-related stress for all individuals, as well as to properly equate the strengths of these processes, it seems that in general effects of workload on physiology are less strong than effects of emotional stress (Harris, 2001; Hofmann et al., 2006). Future experiments using the Sing-a-Song Stress Test could dissociate workload and emotional stress to some extent by telling participants which well-known song they are supposed to sing or by replacing the neutral messages by neutral mental tasks. Also, overall mental stress may be increased by increasing the workload aspect of the task (e.g., “sing a song that is about a city and that none of the participants before you have sung”).

Stress responses when repeating the paradigm in the same individual could reveal to what extent there is habituation. If stress can be repeatedly induced, this would increase the value of the test for fMRI studies. For the TSST and other stress protocols, habituation of the neuroendocrine processes has been reported but the sympathetic nervous system has been found to respond more uniformly to repeated exposure to psychosocial challenge (Kudielka et al., 2007).

Future studies that may help to further improve the paradigm include examining the effect of the number of confederates (with a minimum of zero—where the experimental leader is the only audience available) as well as the effect of the role they play. Now we had confederates waiting in the waiting area and fill out forms so as to make the participants believe that they were really fellow participants, but this may not be necessary. Four of the 25 participants indicated after the experiment that they had doubted that the confederates were real participants. While obviously, this number of participants is too low to allow firm statements on the possible effect of believing the scenario, these four participants did not show a trend to weaker physiological or subjective stress responses. Another way of improving the paradigm could be to reduce the number of neutral messages. Figure 2 shows that there is not much heart rate and skin conductance variation over the first nine blocks suggesting that e.g., four rather than nine blocks may be sufficient. Finally, future experiments should include a baseline subjective stress rating before the experiment starts, and not only after the stress interval. While the time of baseline would be different than the physiological baseline at block 9, taking the difference between the baseline and the subjective rating about experienced stress in block 10 would remove the effect of differences between participants in rating own subjective stress in general. This difference measure would probably comprise a more sensitive subjective stress measure that is expected to correlate better with skin conductance and heart rate responses.

### Detecting sudden increase in stress: applications

This study provides a clear and well-controlled example of the strong effects of mental stress on heart rate and skin conductance. We implemented simple algorithms that could be used in real time to identify a sudden increase in stress within an individual and showed that this was also possible, be it to a lesser extent, using only a camera based heart detection system. This may enable potential applications. Examples are the evaluation of interventions (e.g., scents) that reduce the stress inducing quality of certain stimuli (e.g., a dentist drill) or supporting a therapist when administering anxiety-evoking stimuli in exposure therapy (Popovic et al., 2006). Applications of stress detectors would be particularly valuable in cases where individuals cannot or do not want to express the stress they experience. This could be the case in patients who cannot express themselves effectively due to physical or mental limitations. Stress detectors may help to clarify how they experience certain medical treatments and how they can be modified to elicit less stress in general or in a particular individual. In the safety domain, stress detectors (from a distance) may be one way to increase the chances of picking the appropriate individuals from a waiting line for search by looking at responses to stimuli like the appearance of a drugs sniffing dog. Of course, many of these situations do involve potential effects of movement that we here carefully excluded. These effects will give rise to false alarms. False alarm rates could be reduced by taking into account concurrent measurements of movements. It may also be necessary to limit application of such a stress monitor to situations where there are very little movements. Regardless, in any application the presence of false alarms should be dealt with, e.g., by treating the system as one of several (fallible) stress indicators, or, in the case of evaluating interventions, by looking at the mean of large data sets. Not discussed here, but essential when designing applications around mental stress detection are privacy concerns.

### Conclusions

By making use of the relatively strong effects of social evaluative threat on physiological measures of stress, we designed a new experimental paradigm to induce stress at a clearly specified moment in time in an easy and effective way. The effects of heart rate and skin conductance turned out to be strong and detectable on the level of an individual participant. We hope that the Sing-a-Song Stress Test will facilitate standardized studies on mental stress that are controlled for sensory and movement confounds.

### Conflict of interest statement

The authors declare that the research was conducted in the absence of any commercial or financial relationships that could be construed as a potential conflict of interest.
